# Bone wax can lead to foreign body reaction and local osteolysis after open femoroacetabular impingement (FAI) surgery

**DOI:** 10.1007/s00402-025-05821-z

**Published:** 2025-04-05

**Authors:** Christiane Sylvia Leibold, Andreas Hecker, Florian Schmaranzer, Klaus Arno Siebenrock

**Affiliations:** 1 Department of Orthopaedic Surgery and Traumatology, Inselspital, Bern University hospital, University of Bern, Freiburgstrasse, Bern, 3010 Switzerland; 2 ArthroClinic Bern, Lindenhofspital, Bremgartenstrasse 117, 3012 Bern, Switzerland; 3 Department of Diagnostic-, Interventional-, and Pediatric Radiology, Inselspital, Bern University Hospital, University of Bern, Freiburgstrasse, Bern, 3010 Switzerland; 4 Faculty of Medicine, Department of Radiology, Balgrist University Hospital, University of Zürich, Forchstrasse 340, 8008 Zürich, Switzerland

**Keywords:** Bone wax, Foreign body granuloma, Osteolysis, Offset correction, Femoroacetabular impingement

## Abstract

**Introduction:**

Bone wax is a haemostatic agent, widely used to prevent bleeding from bone surfaces. Despite its effectiveness in haemostatic control, it can lead to foreign body granuloma and osteolysis. Therefore, the aim of this study was to assess the rate and progress of osteolysis after surgical bone wax application.

**Methods:**

We included 425 patients between 01/2002 and 12/2006 that underwent offset correction for cam type femoroacetabular impingement with application of bone wax for homogeneous statistical cohort formation. Comparison was made to a similar cohort group undergoing offset correction without application of bone wax, including 479 patients between 01/2008 and 12/2012. Out of the study group, six hips in five patients presented with persisting pain and growing osteolysis on the X-rays in the area of the offset correction, and two underwent subsequent revision surgery. None of the patients in the cohort group presented with osteolysis. In both groups, patients who presented with persisting pain without radiological osteolysis had other determinable causes as labral tears, progressing osteoarthritis, trochanteric bursitis, and adhesions as suggested source of the pain. We measured the relative area of the osteolysis where present (area of osteolysis/area of femoral head in %) on lateral radiographs on the first postoperative X-rays and latest follow-up X-rays, with a mean follow-up time of 8.6 ± 2.5 years (range, 5–13 years). Histologic samples were taken at revision surgery.

**Results:**

The relative area of osteolysis increased in all hips from a directly postoperative median of 5.5% ± 2.7% (2.3-10.7%) to 11.2% ± 3.9% (7.1-17.3%) at last follow-up. In patients undergoing revision surgery for osteolysis, remaining wax as a foreign material with attached multinucleated giant cells and abundant mononuclear cells was detected histologically.

**Conclusion:**

The intra-articular use of bone wax should be approached with caution and with awareness of the possible complications.

**Trial registration number:**

KEK 2018-00078, registered April 2018.

**Level of evidence:**

level IV, retrospective case series.

## Introduction

Bone wax is a hemostatic agent, which is known to be effective in the prevention of bleeding from bony surfaces [28]. Due to its effectiveness in haemostatic control, bone wax has found widespread use in several surgical disciplines (e.g. orthopaedic surgery, neurosurgery, thoracic surgery, ophthalmologic surgery and maxillofacial surgery). Though bone wax achieves good bony haemostasis, complications after its use have been reported. Foreign body granuloma as a complication of non-absorbable bone wax has not only occured in orthopaedic surgery [6, 21] but also in thoracic surgery [25], neurosurgery [19, 20], ophthalmologic surgery [12, 15] and oral surgery [11, 26]. All these disciplines reported cases of foreign body granuloma and bone wax-associated complications such as non-union and infections of sternotomies [25], nerve compression [11, 12, 15] and inflammatory reactions due to foreign body granuloma [6, 9, 15, 21, 26]. Osteolysis has not been reported as a complication of bone wax in the literature so far, but it is a known complication of foreign body granuloma and inflammatory reaction [24].

In surgical hip dislocation for femoroacetabular impingement surgery, non-absorbable bone wax, consisting of beeswax, paraffin and isopropyl palmitate has been used to stop the profuse bleeding which usually occurs after resection of the cam deformity at the femoral head-neck junction [16]. Even though there are known complications of the surgical hip dislocation (trochanteric bursitis with 12.5%, heterotopic ossifications grades 1–4 according to Brooker with 6.8%, adhesions with about 6%, wound haematoma/infection with about 2% and trochanteric fixation failure with 1–1.8% [22]), osteolysis has not been one of the mentioned complications until now. As we observed focal osteolysis after the application of non- absorbable bone wax in some of our orthopaedic patients after surgical hip dislocation for femoroacetabular impingement surgery, we wanted to assess the rate and progression of osteolysis after the application of bone wax.

We therefore asked (1) what is the rate of osteolysis and (2) is there a progression of osteolysis over time?

### Patients and methods

We performed a retrospective case series on patients after offset correction due to cam-type femoroacetabular impingement. 

Impingement, notching, infection, and metal corrosion are known causes of osteolysis. In patients after successful correction of femoroacetabular impingement, none of these causes are usually present. Therefore, we chose this patient group for analysis to prevent bias from other causes of osteolysis. Every observed osteolysis in these patients was considered to be related to bone wax. We did not include other surgical techniques, indications or surgical sites to provide statistical comparability. Our surgical technique consisted of a surgical hip dislocation with a digastric trochanteric osteotomy, performed in the lateral decubitus position. The cam-type femoroacetabular impingement was then addressed with a round high-speed burr and, in the earlier years (first study group) non-absorbable bone wax was applied on the bleeding bone at the area of the offset correction. The control study group underwent the same procedure but without application of bone wax. The use of non-absorbable bone wax was the in-house standard at the time when the first patient group was operated on but was later abandoned.

We included all patients from our institutional database that underwent offset correction at femoroacetabular impingement surgery between 01/2002 and 12/2006, with at this time routinely performed application of non-resorbable bone wax (Ethicon, Johnson & Johnson, New Brunswick, New Jersey US) and compared them with a second group who underwent offset correction at femoroacetabular impingement surgery without application of non-absorbable bone wax at our orthopaedic department between 01/2008 and 12/2012, after we abandoned the routine application of bone wax for this procedure in 2007. The local ethics commission approved the study (KEK 2018-00078). All patients provided oral informed consent.

We identified 571 patients in the group with the application of bone wax. Of those patients, 146 had to be excluded due to incomplete follow-up, previous operations, incomplete offset correction or trauma, which could lead to bias regarding the cause of osteolysis. In total, we included 425 patients in the group with bone wax application. The patients had a mean age of 29 ± 9 (20 - 48) years at offset correction. In the second group without the application of bone wax, we identified 617 patients. Of those patients, 138 had to be excluded due to incomplete follow-up, previous operations, incomplete offset correction, or trauma. The included 479 patients had a mean age of 27 ± 10 (16 - 44) years at offset correction. Cross-table lateral hip radiographs were obtained according to a standardized technique. The patient was positioned supine with the leg internally rotated, using a film-focus distance of 1.2 m, and with the central beam directed at the inguinal fold. The mean total X-ray follow-up was 8.6 ± 2.5 years (5-13 years) for the study group and 8.4 years ± 2.4 years (5-12 years) for the comparison group. To determine the rate of osteolysis, we reviewed all postoperative X-rays of the included patients in both groups. We defined an osteolysis as a loss of bone on follow-up X-rays compared to the initial post-op x-rays in the area of the offset correction. To assess any changes in the size of the focal osteolysis, we measured the relative area of the osteolysis as a quotient in % (area of the osteolysis in mm² / area of the femoral head in mm² *100) on the standardized cross-table lateral hip radiographs with OsiriX (Pixmeo, SARL, Bernex, Switzerland). The programme calculated the measured area in mm² based on the outlines of the area, which were defined and controlled by two of the authors. We then compared the first quotient after the operation with the quotient from the last follow-up. We used a quotient with the femoral head as a size reference, as the images were not all calibrated at this time.

Statistical Analysis was performed with SPSS Version 1.0.0.1406 using the Wilcoxon paired test and the Fisher´s exact test for statistical testing. Statistical significance was set as a < 0.05.

In case of revision surgery due to progressive osteolysis, histological samples of the femoral head were taken intraoperatively.

## Results

Osteolysis was found in 6/425 (1%) cases in the group with the application of bone wax and in none 0/479 (0%) of the patients of the comparison group without bone wax application (Table 1). This difference was found to be significant, with a p-value of 0.0106, by using Fisher´s exact test. Detection of osteolysis was evaluated using Cohen’s Kappa for intra- and interobserver reliability and produced a strong coefficient (0.88) for intraobserver reliability and a good coefficient (0.74) for interobserver reliability.

**Table 1:** Case demographics and measurements

Case (pseudonym)	Age at initial surgery (years)	Area of osteolysis initially postoperative (% of femoral head)	Area of osteolysis at last Follow up (% of femoral head)	Growth Osteolysis (%)	Follow up (years)
A	20.0	10.7	17.3	61.4	8
B	32.9	5.4	11.1	104.8	10
C	26.9	3.4	7.1	108.1	11
D	24.8	4.7	7.6	63.3	5
E	25.8	2.3	8.6	281.1	5
F	48.3	6.8	15.6	129.1	13
MEAN	29.8	5.5	11.2	124.6	8.6

All six of the cases with osteolysis had persistent pain, and two needed revision surgery due to the growing osteolysis and persistent pain. We found the patients with osteolysis on the X-rays to have a presentation with dull pain with movement and while resting. The pain was described differently to the previous preoperative pain due to impingement and was first described in 3 of the hips after 3 months. It was present in all six hips after one year after the operation. One revision was a hip arthroscopy with debridement because a soft tissue mass was forming in the area of the osteolysis, and the other was a conversion to a total hip prosthesis as the osteolysis was expanding into the cartilage area and the patient chose a hip replacement after a discussion of the surgical possibilities. In the latter, the femoral head was taken for histopathological examination.

**Fig. 1:**
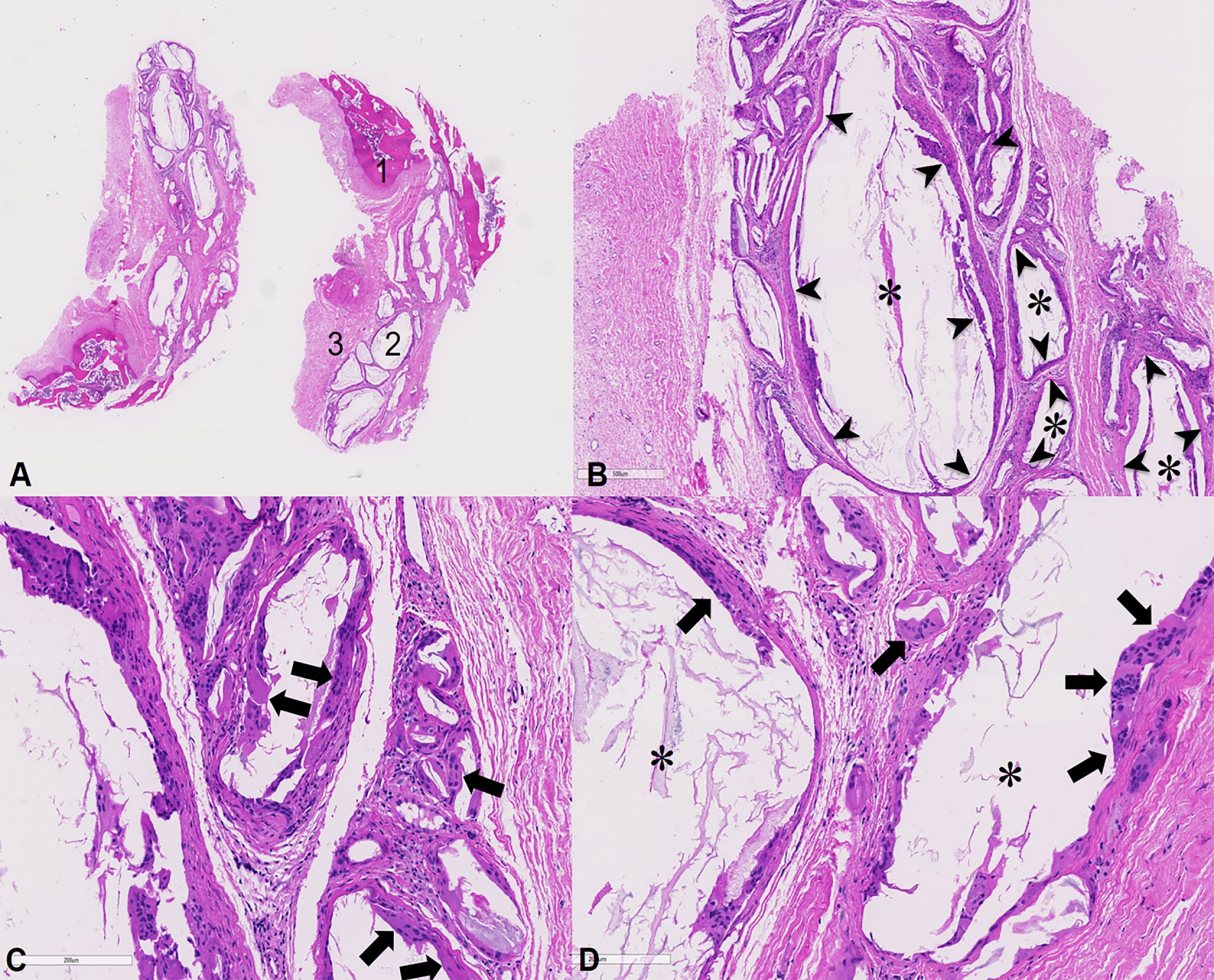
2 **A**: Light-optical microscopy of histopathological sample of area of offset correction of patient C after 9 years after offset correction at conversion to THA at 10x magnification. Haematoxylin-Eosin staining and 3 μm slices of zone of offset correction 9 years postoperatively. (1=bone, 2=bone wax, 3=fibrous tissue) **B**: Histopathological light-optical microscopy at 20x magnification with Haematoxylin-Eosin staining and 3 μm slices of zone of offset correction 9 years postoperatively. (Asterisk: bone wax, arrowheads: foreign body granuloma around bone wax) **C** and **D**: Histopathological light-optical microscopy at 40x magnification with Haematoxylin-Eosin staining and 3 μm slices of zone of offset correction 9 years postoperatively. Typical polynuclear giant cells (arrows) surrounding the bone wax (asterisk)

**Fig. 2:**
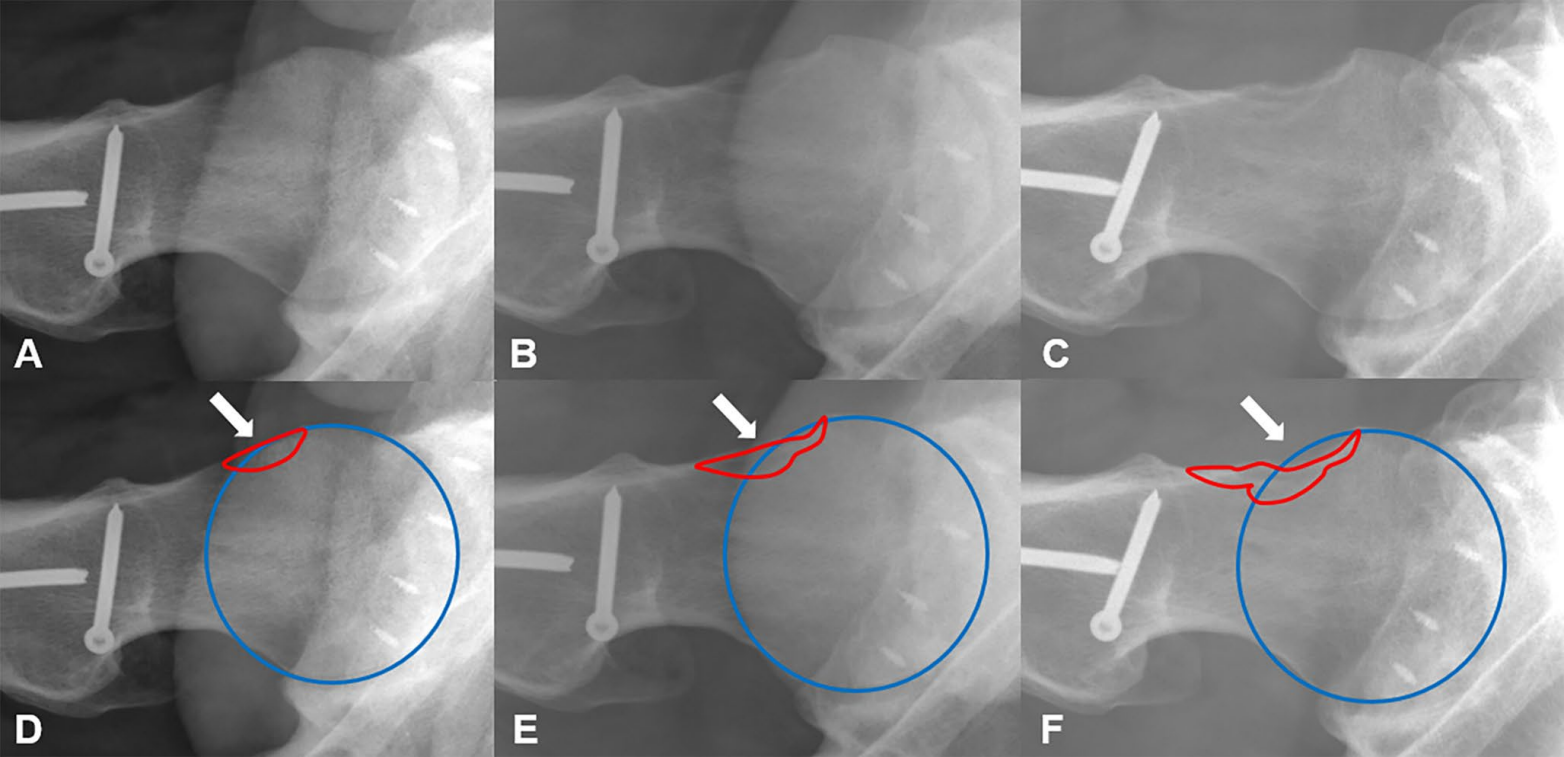
Comparison of area of femoral head and osteolysis over the years. **A**: Initial X-ray after offset-correction, **B**: X-ray five years after offset correction, **C**: X-ray 10 years after offset correction, **D**: X-ray after offset-correction with measurements of area of femoral head (blue) and initial area of offset correction (red), **E**: X-ray five years after offset correction with measurements of area of femoral head (blue) and osteolysis (red), **F**: X-ray 10 years after offset correction with measurements of area of femoral head (blue) and osteolysis (red)

There, we found bone wax persisting as a foreign body 9 years after application at femoroacetabular impingement surgery. The bone wax particles were surrounded by foreign body granuloma with the typical cell findings of polynuclear giant cells, different types of mononuclear cells and fibroblasts, consistent with aseptic osteolysis. There were no other unusual findings which could explain the osteolysis (Fig. 1A-D). (2) In all patients with osteolysis, the growth of the osteolysis was well visible on the follow-up x-rays, e.g. (Fig. 2).

**Fig. 3:**
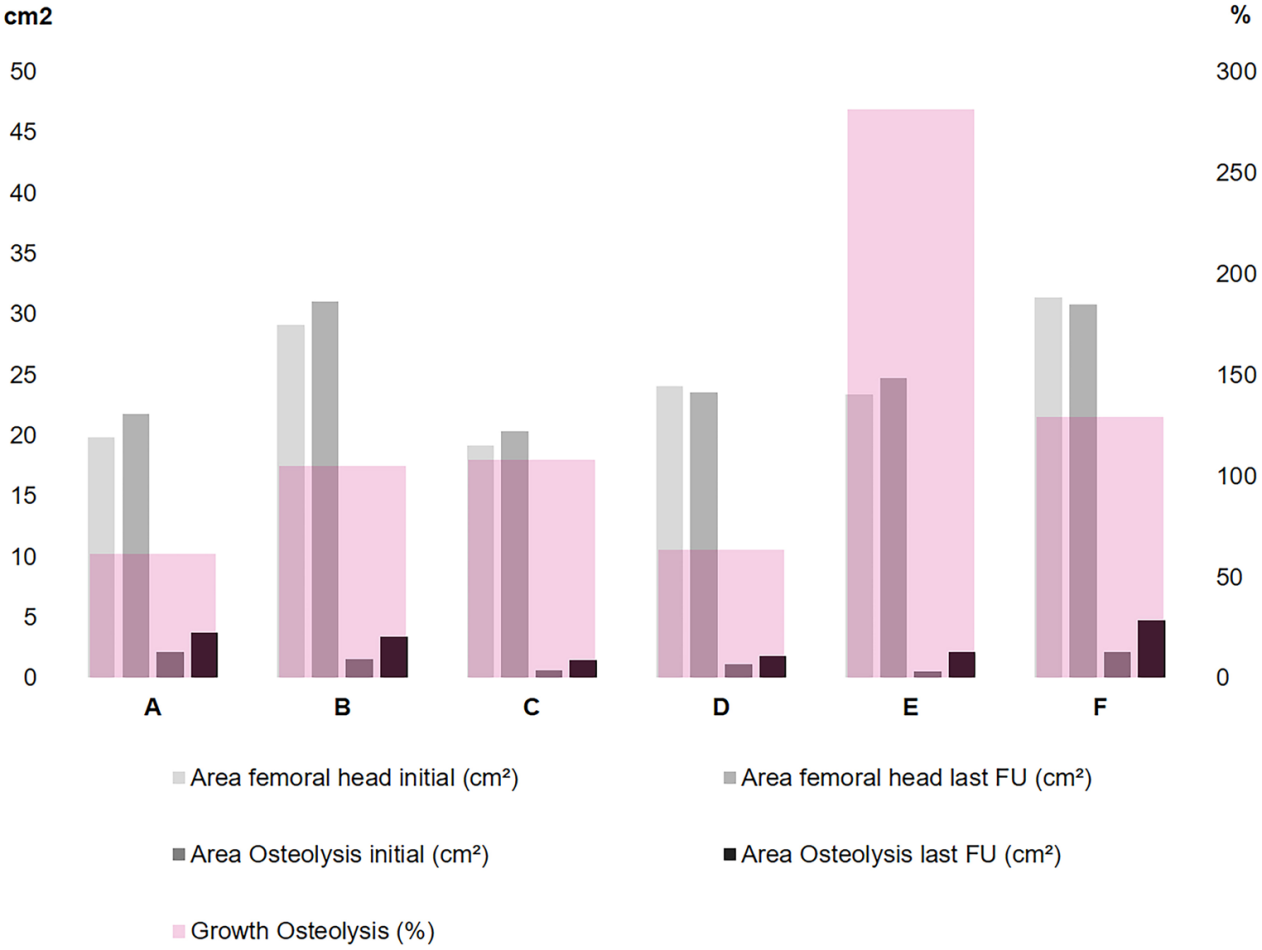
Comparison of measurements for all patients (named **A**-**F**) of the area of the femoral head at initial and latest follow-up, the measurements of the area where the osteolysis develops at initial and latest follow-up, and the growth of the osteolysis in percent comparing initial and last follow-up

The quotient of the radiographic measurements (area of osteolysis/area of femoral head) showed a growth of the osteolysis in all six cases when comparing initial postoperative and latest follow-up X-ray (Fig. 3). The mean initial quotient, displaying the amount of the area of the offset correction as percentage of the femoral head was 5.5% ± 2.7% (2.3-10.7%), the mean quotient at the latest follow-up was 11.2% ± 3.9% (7.1-17.3%). Comparing the values, the mean increase of percentage was 124.6% ± 74.1% (61.4– 281%), meaning that the defect in the area of offset correction has more than doubled. There was a significant statistical difference comparing the initial quotient and at latest follow-up (*p* = 0.02). Both in the bone wax group and in the control group, other patients also reported ongoing pain after the operation, but none of them showed signs of osteolysis. All those patients had other identifiable causes for the pain (progressing osteoarthritis, heterotopic ossifications, trochanteric bursitis, and adhesions). Screw removals not counted as revision, we had a 10% revision rate in the bone wax group versus a comparable 10.5% revision rate in the comparison group (Table 2). Reasons for revision were: Arthroscopic adhesiolysis with a 6% revision rate in the bone wax group and 6.5% in the comparison group, evacuation of wound haematoma with 1.5% in the bone wax group and 2% in the comparison group, wound infection with 1% in the bone wax group and 0.5% in the comparison group, trochanteric fixation failure with 1% in both groups, and heterotopic ossification Grade 4 (Brooker) with 0.5% in the comparison group. The revisions in the bone wax group due to the growing osteolysis and pain accounted for under 1% of revisions in the bone wax group.

**Table 2:** Comparison of complications requiring revision between patients with and without the application of bone wax

Reason for revision	Prevalence among patient group with application of bone wax (%)	Prevalence among patient group without application of bone wax (%)
Adhesions	6	6.5
Wound haematoma	1.5	2
Wound infection	1	0.5
Trochanteric fixation failure	1	1
Heterotopic ossification	0	0.5
Miscellaneous	< 1	< 1

The performed revisions generally consisted of arthroscopic removal of adhesions, re-osteosynthesis of trochanteric osteotomy, wound revision, and removal of heterotopic ossification.

## Discussion

In this retrospective case series analyzing the effect of non-absorbable bone wax application during femoroacetabular surgery, we found an occurrence of osteolysis in 1% of the cases with bone wax application. The osteolysis was growing during the observational period of a mean time of 9 years from a mean initial value of 1,3 cm2 to a mean value of 2,9 cm2 at the last follow-up. Histologically, we found a foreign body reaction at revision surgery in one case, 9 years after bone wax application.

There are several reports about foreign body granuloma after bone wax application from different surgical disciplines. Foreign body granuloma have been reported after bone wax application at alveolar surgery (Aurelio et al. [3]); at the iliac crest (Faghahati [6]); at foot surgery (Hill et al. [9]); after molar surgery causing alveolar nerve damage (Katre et al. [11]); compressing the optic nerve in the orbit (Katz et al. [12]); after offset correction (Lavigne et al. [15]); after laminectomy at the lumbar spine in the disc space (Ozdemir et al. [19]); at the cerebellopontine angle (Patel et al. [20]); and at a cranial defect (Wolvius [26]), but no study has reported osteolysis as a side effect of bone wax yet, though studies exist, that suggest bone wax can suppress new bone formation in the resection area [25]. Vestergaard et al. showed in a porcine model that bony healing was delayed, and fracture strength and calcification were lower after the application of bone wax compared to application of a water-soluble polymer wax. Looking at the findings of foreign body reaction and osteolysis in the context of basic research, a possible relationship between the two phenomena can be assumed. A foreign body reaction leads to a high number of multinucleated foreign body giant cells, macrophages, as well as to the expression of collagenases and proteases [7, 10]. Synovial tissue seems to be prone to generate this reaction. In addition, a generalized synovitis with lympho-plasmatic infiltration was reported in other studies [5, 18, 21]. Especially the macrophages release pro-inflammatory mediators like prostaglandins and many others after phagocytosis of foreign bodies [1]. This inflammation can finally lead to chondrolysis, bone resorption, and end stage osteoarthritis [8, 17]. Proteases released during this inflammatory process are mainly responsible for damaging the cartilage [27]. Moreover, a constant phagocytic reaction and release of lytic enzymes eventually results in constantly increasing osteolysis through osteoclast activation [13]. The final result is a fulminant inflammation with cartilage and bone destruction [23]. The bone loss is also a result of a high count of osteoclasts, which differentiate from macrophages [14]. Given that macrophages are very important in the genesis of destructive osteoarthritis and the fact that they are found in very high counts in foreign body reactions, it suggests that those types of cells have osteodestructive effects, which can cause osteolysis [2, 4]. According to the literature, the clinical presentation of foreign body granuloma ranges from incidental findings to persistent pain. In our study group with the application of bone wax, we found the patients with osteolysis on the X-rays presented with dull pain during movement and while resting. Both in the bone wax group and in the control group, also other patients reported ongoing pain after the operation, but none of them showed signs of osteolysis. Additionally, all those patients had other identifiable causes for the pain (progressing osteoarthritis, labral tears, trochanteric bursitis, and adhesions). The clinical consequence of osteolysis in the neck of the femur after offset correction seems to be, first and foremost, pain with exercise and at rest. A progressive osteoarthritis due to cartilage and bone damage seems likely. A higher risk for femoral neck fractures, especially in case of trauma, might be possible for osteolysis located in the femoral neck area as in this study. Overall, the clinical consequence of osteolysis due to bone wax is hard to determine and seems to be a rare complication. However, bone wax is widely used at different locations in orthopaedic surgery, and other surgical specialties use bone wax in various indications but for the same purpose of local hemostasis. Therefore, we assume that our results also apply to other operations, surgical sites, and specialties. Bone wax can be divided into absorbable and non-absorbable types [26]. When comparing the literature, most of the reported complications occurred with the usage of non-absorbable bone wax or were not classified further. We acknowledge limitations to this study. First, it is a retrospective case series with the observation of a rare complication. Second, the radiographic imaging of the osteolysis was done by X-rays and not by 3D imaging at that time and therefore has a lower accuracy than 3D imaging, such as a CT scan might have had.

Furthermore, we were only able to obtain a histopathological sample of one of those six hips. Therefore, we cannot say that the other five osteolysis we observed were also directly related to a foreign body granuloma. Most of the reported cases of foreign body granuloma in the literature were diagnosed clinically and histopathologically only in cases of revision surgery, so possible osteolysis without complications could have been missed.

## Conclusion

This study suggests that osteolysis due to a long-lasting foreign body reaction might be a rare complication after the application of non-absorbable bone wax during FAI surgery.

Surgeons should be cautious when using bone wax and be well aware of the possible side effects. To avoid this problem, the application of non- absorbable bone wax or the use of other haemostatics should be considered thoroughly. If the type of bone wax used makes a difference remains subject to further investigation.
